# Supplementary material for: Tracking of quiescence in Leishmania by quantifying the expression of GFP in the ribosomal DNA locus
Source: Sci Rep. 2019 Dec 12;9:18951. doi: 10.1038/s41598-019-55486-z (PMC6908629; doi:10.1038/s41598-019-55486-z)
Supplement: Supplementary file 1 — Supplemental information [file 41598_2019_55486_MOESM1_ESM.pdf]

Tracking of quiescence in *Leishmania* by quantifying the expression of GFP in the ribosomal DNA locus

Marlene Jara, Ilse Maes, Hideo Imamura, Malgorzata A. Domagalska, Jean Claude Dujardin, Jorge Arevalo

Supplemental material

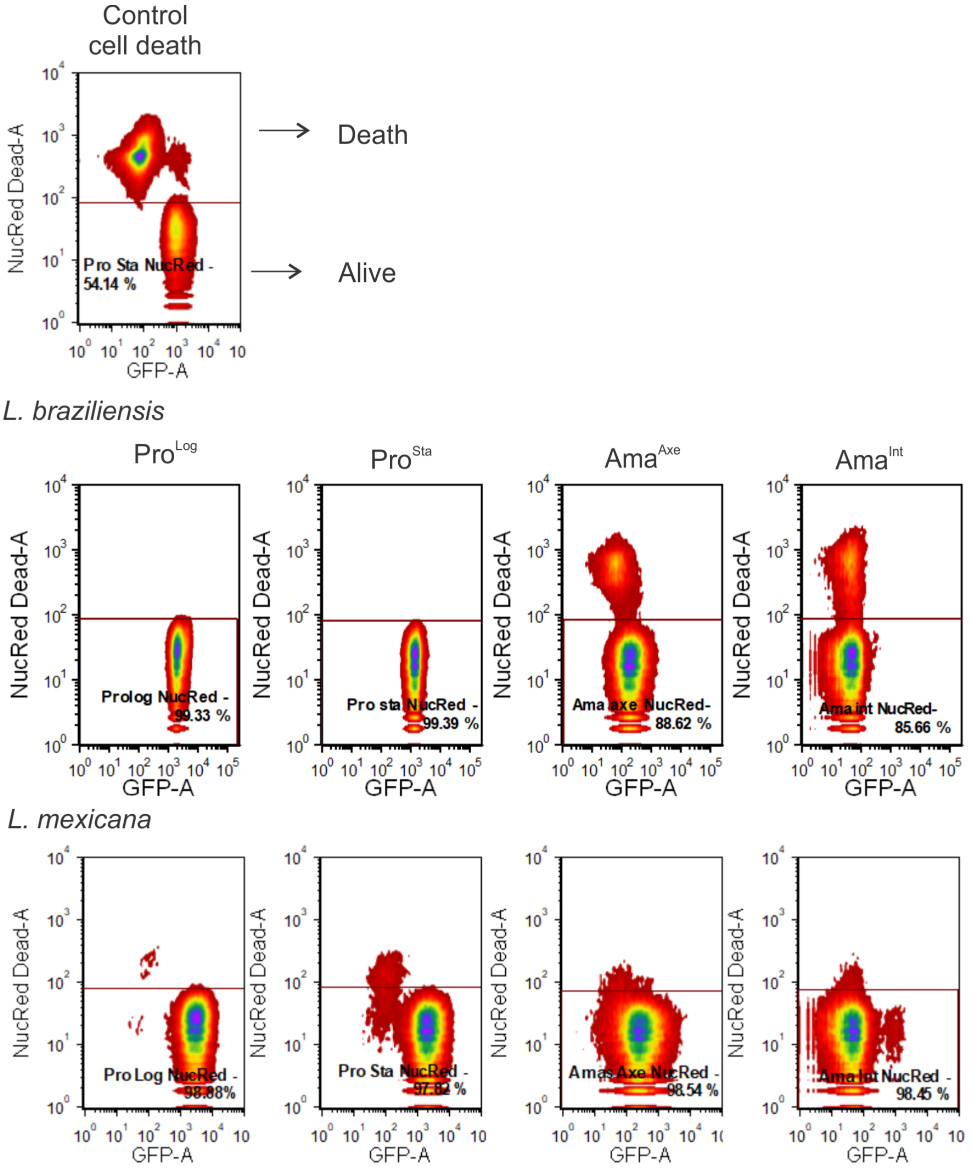

Fig S1. Cell viability of the harvested parasites in both *L. braziliensis* and *L. mexicana*. The parasites were stained simultaneously with the non-permeable fluorophore NucRed Dead, which stains the DNA of dead parasites. A control of dead parasites (stationary promastigotes at day 10) is included. The percentage of viable population is shown on each plot. Each plot represents one of three biological replicates.

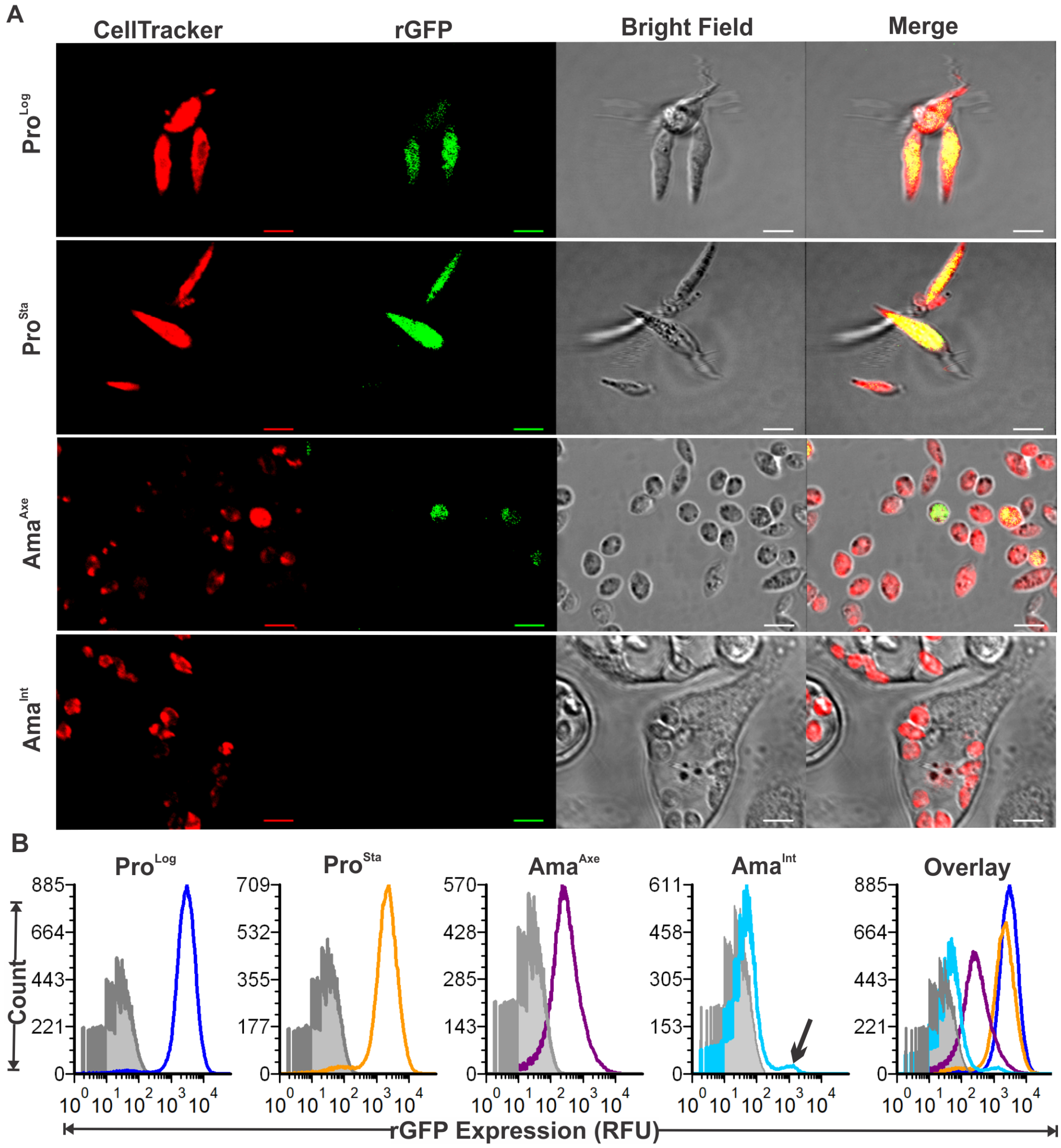

Fig S2. Live cell imaging of rGFP expression in *L. mexicana* Bel21 rGFP Cl1. (**A**) The expression of rGFP was monitored by confocal microscopy in Pro^{Log}, Pro^{Sta}, Ama^{Axe} and Ama^{Int}. As in *L. braziliensis,* a deep downregulation of rGFP expression was observed in Ama^{Axe} and Ama^{Int} of *L. mexicana*. The scale bar in each figure represents 5 µM. **B,** Quantification of rGFP expression over the cell cycle of *L. mexicana* by Flow cytometry. The gray peak in each panel is an overlay of the Wild Type strain (Non GFP negative control). Each histogram represents 10^5 live cells, all negative for the NucRed staining.

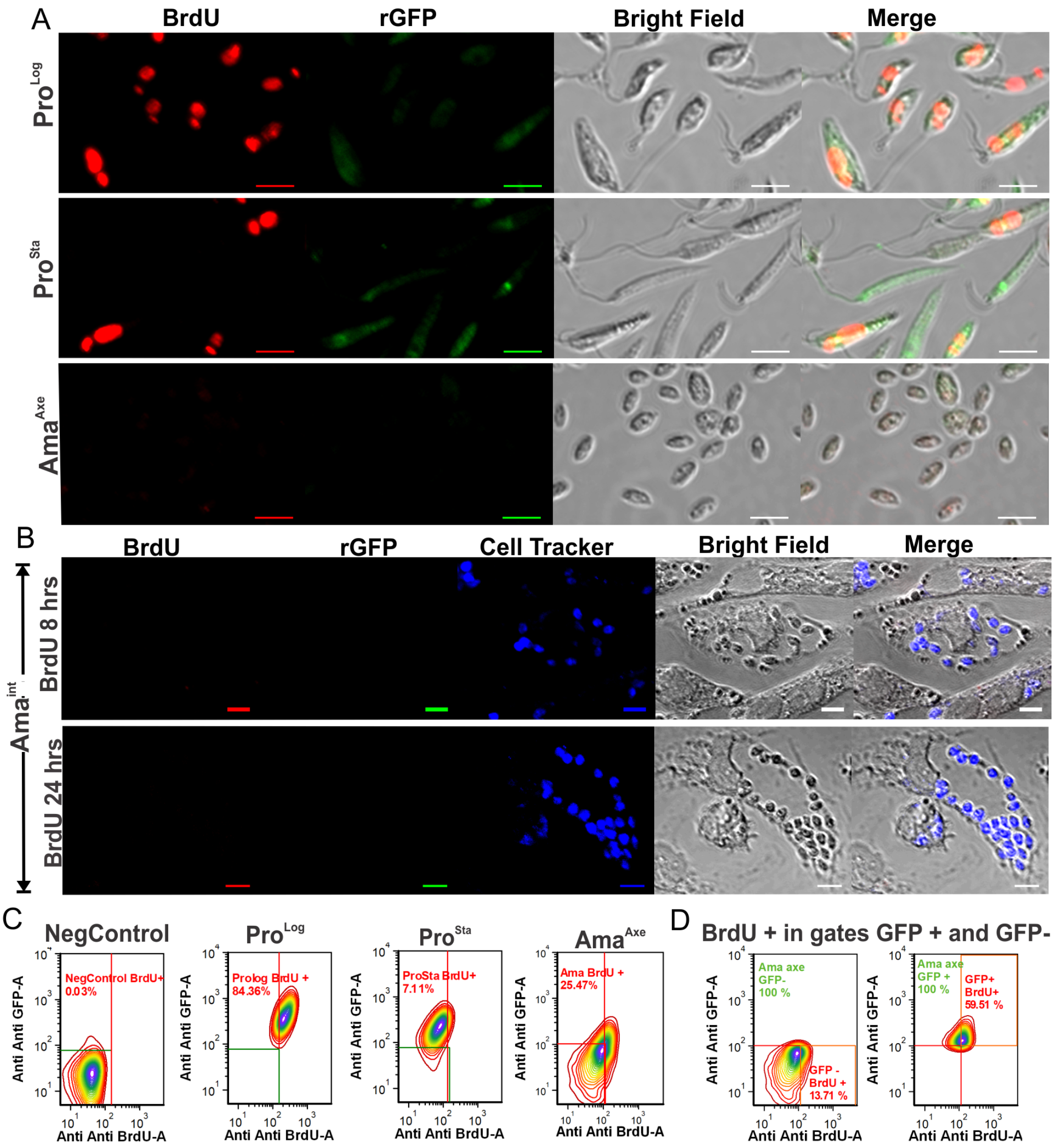

Fig S3. Relationship between BrdU incorporation (as a measure of replication) and rGFP expression in promastigotes and amastigotes of *L. mexicana*. **A**, Immunodetection (confocal microscopy) of rGFP and BrdU, 8 hrs after exposure with BrdU in Pro^{Log}, Pro^{Sta} and Ama^{Axe}. **B**, Immuno-detection of BrdU and rGFP in Ama^{Int}. In order to be able to monitor Ama^{Int} inside the parasitophorous vacuole the parasites were pre-stained with Cell Tracker before the infection. The scale bar in each picture represents 5 µM. **C,** Quantification by flow cytometry of the percentage of cells in a proliferative stage as evidenced by the incorporation of BrdU after 8 hrs of incubation. D, Quantification of the percentage of proliferative cells in the subpopulation GFP^{+} and GFP^{-} in axenic amastigotes after 8 hrs of incubation with BrdU. The fraction of $BrdU^{+}$ cells was ~ 5-fold higher in the GFP^{+} subpopulation in comparison to the GFP^{-} subpopulation.

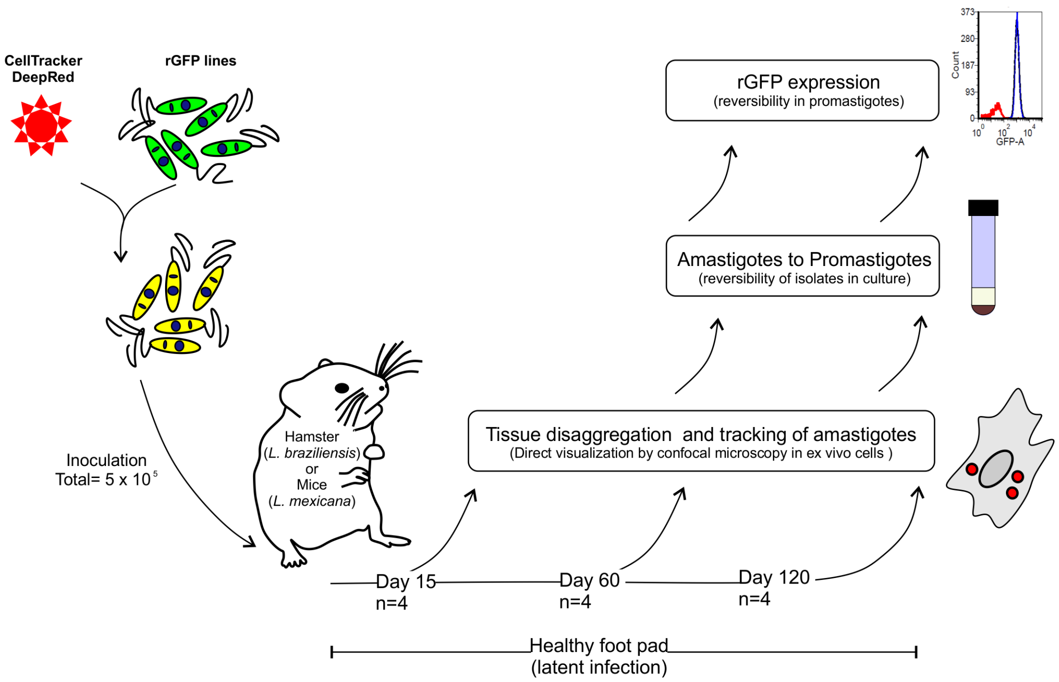

Fig S4. Depiction of the animal experimental work for the evaluation of rGFP expression in disaggregated rodent tissues post infection with *L. braziliensis* and *L. mexicana.* Twelve animals were inoculated in the foot pad with 5 x 10^5 pre-stained stationary promastigotes. On days 15, 60 and 120 the animals were euthanized and the foot pad were collected in the absence of lesions or foot pad swelling (latent infection). Direct tracking of the amastigotes within the cells from the disaggregated foot cells was done by confocal microscopy with settings for the codetection of the CellTracker DeepRed and GFP. On days 60 and 120 an aliquot of the cells was placed in Tobie blood agar in order to confirm the presence of amastigotes. After the propagation as promastigotes the presence and upregulation of rGFP expression was performed by flow cytometry.
